# Changes in Turkish- and Resettler-origin Adolescents’ Acculturation Profiles of Identification: A Three-year Longitudinal Study from Germany

**DOI:** 10.1007/s10964-020-01250-w

**Published:** 2020-05-13

**Authors:** Philipp Jugert, Sebastian Pink, Fenella Fleischmann, Lars Leszczensky

**Affiliations:** 1 Department of Psychology, University of Duisburg-Essen, Essen, Germany; 2 MZES, University of Mannheim, Mannheim, Germany; 3 ERCOMER, Utrecht University, P.O. Box 80.140, 3508 TC Utrecht, The Netherlands

**Keywords:** Ethnic identification, National identification, Ethnic minority adolescents, Latent profile analysis, Latent transition analysis

## Abstract

Little is known on how ethnic minority adolescents develop acculturation profiles of identification (i.e., how they combine their ethnic and national identification, such as being high on both and thus rather “integrated” or high on ethnic and low on national and thus rather “separated”). In a first step, this 3-year longitudinal study classified Turkish (*n* = 344) and resettler-origin (*n* = 121) ethnic minority adolescents living in Germany (*M*_age_ = 14.2, SD = 1.54, 51.6% female) according to their levels of ethnic and national identification. Latent profile analyses identified four profiles (separated, integrated, medium-ethnic, low-ethnic) for the former and three profiles (separated, integrated, low-and-medium ethnic) for the latter group. Latent transition analyses revealed considerable instability of profile attributions over time. Integration declined among both groups and results provided no evidence that national group boundaries are more permeable for resettler-origin than for Turkish-origin adolescents. Additional analyses revealed that perceived ethnic discrimination affected the probability to be in a particular profile but did not moderate transition probabilities. Overall, results suggest that during early-to-mid adolescence it is increasingly difficult to uphold a dual identity.

## Introduction

Identity formation is an important developmental task during adolescence. Ethnic minority adolescents face the additional acculturative task of finding ways to balance two important identity domains—their ethnic and their national identity. This involves developing a sense of belonging, affection, and pride to their ethnic community and heritage culture as well as to their country of residence. There are different ways how these identifications can be combined (e.g., being high on both or being high in ethnic and low in national identification) and these identification profiles are in turn differentially related to social-emotional wellbeing and adjustment (Nguyen and Benet-Martinez 2013). Yet, little is known about how ethnic minority adolescents develop a particular identification profile and how stable these profiles are. Unfortunately, most previous research has used cross-sectional designs, limiting the understanding of the inherently developmental and dynamic process of acculturation. The aim of this study therefore is to examine the development of acculturation profiles of identification among the two largest groups of ethnic minority adolescents in Germany—Turkish-origin and ethnic German resettler-origin youth from the former Soviet Union. While all immigrants and their descendants face high pressures for assimilation in Germany (Zick et al. 2001) the social climate is more welcoming for resettler-origin youth than for Turkish-origin youth and thus the conditions for the development of acculturation profiles of identification are quite different for these two groups (Schotte et al. 2018).

Ethnic and national identification can be mapped onto Berry’s (1997) bidimensional model of acculturation, which refers to orientations toward ethnic (heritage) culture and national (host) culture. While these acculturation orientations comprise practices, identifications, and values in relation to both cultures (Schwartz et al. 2010), the focus is on identification here. When the strength of ethnic and national identification is independently measured and crossed, this results in four different acculturation profiles of identification: integration (ethnic and national identification high), assimilation (ethnic identification low—national identification high), separation (ethnic identification high—national identification low), and marginalization (both identifications low). Integration (also referred to as dual identification or biculturalism) is often associated positively with psychological adjustment, wellbeing, school achievement, and civic engagement (Nguyen and Benet-Martinez 2013), though this may depend on the sociopolitical context (i.e., pressure for assimilation), the particular ethnic minority group, and the outcome domain (Schotte et al. 2018). But how do ethnic minority adolescents come to adopt a particular acculturation profile of identification, and how does it change over time?

### The Development of Acculturation Profiles of Identification

While ample research has examined the links between particular acculturation profiles of identification and measures of psychological and sociocultural adjustment, there is a lack of both empirical evidence and theorizing on how adolescents develop a particular acculturation profile of identification in the first place (c. Schwartz et al. 2018). A rich literature on ethnic-racial identity, however, suggests that ethnic identity development starts already in childhood and continues into adulthood (Umaña-Taylor et al. 2014). Research on ethnic identity is rooted in the model by Marcia (1966), which assumes that identity development consists of exploration and commitment (i.e., identification). Ethnic identity exploration is particularly salient during adolescence when adolescents try to answer the questions of “Who am I?” and “Where do I belong?” (Umaña-Taylor et al. 2014). Individuals commit to an ethnic identity through exploring their ethnic background and learning about the culture, history, and traditions of their ethnic group (Syed et al. 2013). Accordingly, commitment to a particular ethnic identity follows from a phase of exploration, and research shows that changes in ethnic identity commitment are most common between early to middle adolescence when youth are most active in their ethnic identity exploration. Thus, between early and middle adolescence most ethnic minority adolescents show increasing levels of commitment with their ethnic identity (Huang and Stormshak 2011) while ethnic identity commitment remains stable between middle and late adolescence (Pahl and Way 2006). These findings suggest that early to middle adolescence is a key period to study ethnic identity development.

However, research on ethnic identity development is relatively silent about national identity. Research on national identity development has mainly focused on ethnic majority children (Barrett and Oppenheimer 2011), and only recently studies have begun to examine national identity development among ethnic minority adolescents. For instance, one study found a slight downward trend in the strength of national identification among ethnic minority youth in Germany across early adolescence (Fleischmann et al. 2019). Examining a younger age group and shorter time frame, another study, by contrast, found no change in national identification levels among 9–10 year old ethnic minority children in Germany across a 5-month period (Froehlich et al. 2019). A study from the US suggests that national identification may follow a different developmental trajectory than ethnic identity (Kiang et al. 2013). In this study among Asian Americans, ethnic identification stayed stable while American identification increased from middle to late adolescence.

So how do acculturation profiles of identification develop? It has been proposed that first generation adult immigrants will add national identification to an already existing ethnic identification as they bring their ethnic identity with them but are likely to develop a sense of belonging to their country of residence over time (Fleischmann and Verkuyten 2016). However, it is less clear how these multiple identifications develop among adolescents of immigrant origin (often 2nd or 3rd generation immigrants) who go through the phase of identity exploration and commitment as ethnic minority members during adolescence. Many immigrant parents try to instill values of their culture of origin in their children (Suárez-Orozco et al. 2015), and for many immigrant children their own ethnic group therefore often is initially more salient than their country of birth and/or residence (Phinney and Ong 2007b). At the same time, maintaining a high level of identification with two groups (e.g. dual identification) is cognitively more complex than identifying with one group (Roccas and Brewer 2002). The required level of cognitive maturation to maintain an integrated acculturation profile of identification thus might not be achieved before adolescence. Taken together, this suggests that ethnic minority children will first develop an identification with their ethnic group and that national identification will develop later (i.e., during adolescence).

### Previous Research on the Development of Acculturation Profiles of Identification

Little is known about the development of acculturation profiles of identification among early adolescents. A limited number of studies examined developmental trajectories of ethnic and national identification. All of these studies used some form of latent class growth modeling to identify different classes of individuals with similar intercepts and slopes in ethnic and national identification. Studies from the US identified two-class solutions among Mexican-origin (Knight et al. 2009) and Hispanic adolescents (Schwartz et al. 2015). While the former study found one class of high ethnic and low national identifiers (i.e., separated) and another class with moderately high values on both dimensions (i.e., integrated), both classes were characterized by stability across age 14 to 20. The latter study identified two different classes of integration among late adolescents, one with stable identifications, and one with increasing national identification.

Studies from Europe have found more varied trajectory classes. In a German study with resettler-origin youth from the former Soviet Union, three different identification trajectories were identified (Stoessel et al. 2014). One class could be described as assimilated (i.e., high national and low ethnic identification) with increasing levels of ethnic identification, but remaining below the mid-point of the scale. The other two classes could be described as different kinds of integration, one group where individuals start from separated and move to integrated, and another class where both identifications are moderately high and stable over time. Another study with Muslim adolescents in four Western European countries (Spiegler et al. 2019) found four different trajectory classes, integrated (both increasing), moving from separated to integrated, moving from assimilated to integrated, and separated (both decreasing).

All of these studies have provided important insights into the development of acculturation profiles of identification by starting to go beyond the predominantly cross-sectional approaches in acculturation research. Yet, they all identified classes based on average rates of change over time, thus assuming change to be continually occurring at the same rate across class members. But what if change is not continuous (e.g., moving through discrete stages) and more idiosyncratic (e.g., different people taking different paths) and thus reflects qualitative rather than quantitative growth (cf. Perra 2012)? In that case, another analytical approach is needed. This can be achieved by classifying individuals into acculturation profiles of identification using latent profile analysis and then examining transitions between these profiles over time using latent transition analysis (Collins and Lanza 2010). The crucial difference between the latent class growth model approach used by earlier studies and the combination of latent profile and latent transition analysis is that the former focuses on establishing different growth patterns while the latter first tries to establish the number of latent profiles at each time point and then examines whether there is change between latent profiles across time and, if so, how that change can be characterized. As argued by Lee et al. (2018), the latent class growth model approach does not provide a deeper understanding of how acculturation profiles of identification change over time because increasing or decreasing trends in ethnic and national identification do not necessarily imply that individuals switch between profiles. It also does not inform us about whether certain profiles (e.g., integration) increase or decrease across development and what predicts transitions between profiles.

Only one previous study has used a combined latent profile-latent transition analysis approach to examine the development of acculturation profiles of identification (Lee et al. 2018). This study identified two latent profiles of ethnic and national identification among a sample of Hispanic adolescents in the US—low and high integration. Both profiles were characterized by high stability across time, and when changes occurred, individuals were more likely to transition from high to low integrated than vice versa. However, previous research (e.g., Spiegler et al. 2019) suggests that identification profiles are more varied in German samples.

### The Influence of Group Permeability and Ethnic Discrimination

While integration is the most common acculturation profile of identification (Berry et al. 2006), not all ethnic minority adolescents develop a dual identification. Importantly, like all collective identities, ethnic and national identities are social constructions that need to be claimed by the individual and verified by others (North and Swann 2009). This implies that there are social constraints on which acculturation profile of identification adolescents can choose. Thus, members of ethnic minority groups may struggle to develop an integrated profile because members of the dominant society may not want them to retain their ethnic heritage culture (Bourhis et al. 1997). Integration may also be difficult to pursue because the receiving culture may not be willing to grant recognition to ethnic minority members from visible-minority backgrounds as fellow national citizens (e.g., Cheryan and Monin 2005). In the period of (early) adolescence, particularly the ages between 10 and 14, children become aware of stereotypes and the social status position of their group in society (Vedder and van Geel 2017), thus making it a particularly fruitful period to study the development of acculturation profiles of identification.

Social identity theory (Tajfel and Turner 1979) is a useful framework to understand the influence of the social context on the development of acculturation profiles of identification. According to social identity theory, individuals derive positive self-esteem from membership in positively valued groups. However, when groups are low in public esteem as in the case of ethnic minority groups who face discrimination and rejection, this creates a social identity conflict. How this conflict is resolved by the individual depends on structural conditions in society and in particular on the perceived *permeability of group boundaries*. If group boundaries are perceived as permeable, individuals may follow an individual mobility strategy, which would suggest to identify highly with the nation and to dis-identify with the ethnic group (i.e., assimilation; cf. Roccas 2003). If, on the other hand, group boundaries are perceived as impermeable, individual mobility is not an option and therefore separation is more likely. Impermeable group boundaries also make integration unlikely because identifying with two groups is only possible if simultaneous membership in both groups is actually possible.

Research on acculturation distinguishes classic settler societies, which were founded on immigration (e.g., the US, Canada, Australia, New Zealand), from non-settler societies, in which immigration is only recently acknowledged as a social reality (e.g., Europe). In non-settler societies like Germany where national identity is often defined in terms of shared ancestry (Pehrson et al. 2009), it is difficult for phenotypically visible ethnic minority members to be accepted as co-nationals (i.e., group boundaries are rather impermeable). This is particularly the case for Turkish-origin youth in Germany whose national belonging in Germany? is often contested by mainstream members of society (Moffitt et al. 2018). Individuals of Turkish-origin are the largest immigrant group in Germany, comprising around three million people. Turkish immigrants were recruited as so-called “guest workers” for unskilled factory and mining work in West Germany in the 1960s and 1970s. They were not meant to stay but eventually brought their families and settled permanently in Germany. People of Turkish-origin face high levels of discrimination and rejection by non-immigrant members of society (Schaefer and Simon 2020).

By contrast, group boundaries are more permeable for ethnic German diaspora immigrants (so-called “Aussiedler”). These resettler immigrants arrived in the 1990s after the break-down of the Soviet Union. With over 2.5 million individuals, ethnic German resettlers are one of the largest immigrant groups in Germany. They are descendants of ethnically German settlers who had moved to Russia in the 1800s. They have lived in the former Soviet Union for generations and were well adapted to Russian culture (Dietz 2003). Hence, upon arrival in Germany, most of them spoke little German and they have faced typical challenges of all immigrant groups, such as discrimination by host society members and language problems. However, due to their German ancestry, they received preferential treatment by German authorities in form of financial support and immediate citizenship rights. Moreover, ethnic German resettlers are phenotypically White, have mostly German-sounding names, and if they speak German without a Russian accent, they cannot be easily be distinguished from non-immigrants. Support for the notion of different group boundaries for different immigrant groups comes from research on acculturation showing that immigrant groups who are culturally more distinct from non-immigrants typically encounter more acculturative stress and adjustment problems than culturally more similar immigrant groups (e.g., Ward and Kennedy 1993). Research from the US also shows that people from racial minority groups are less likely to be associated with being American than White people (Devos and Banaji 2005). Consistent with the notion of different group boundaries, research shows that compared to immigrants of Turkish origin, ethnic German resettlers are viewed much more favorably by members of the mainstream society (Brüß 2005).

Another context-related factor that is likely to influence the development of acculturation profiles of identification among ethnic minority adolescents is experiencing ethnic discrimination. Being discriminated thwarts developing a sense of belonging to the nation, for it signals that one does not belong to and is not welcome in the national category. In order to protect wellbeing and self-esteem, adolescents may dis-identify with the nation (Jasinskaja‐Lahti et al. 2009) and identify more strongly with their ethnic group (Branscombe et al. 1999). Identifying more strongly with one’s ethnic group in response to ethnic discrimination has been termed reactive identification (Verkuyten 2018) or reactive ethnicity (Rumbaut 2008). It can be seen as a strategic reaction because it seems most fruitful to choose identity options that are actually available to oneself (Schwartz et al. 2018). Past research confirmed that ethnic discrimination boosts ethnic identification (Skrobanek 2009) while decreasing national identification (Fleischmann et al. 2019). Experiences of discrimination have also been found to lead to greater perceived conflict and thus to compartmentalization (i.e., identities are rather separate) between identities (Amiot et al. 2018). Experiences of ethnic discrimination and permeability of group boundaries are related, such that individuals who experience a lot of discrimination may perceive group boundaries to be rather impermeable (cf. Schulz and Leszczensky 2016).

## The Present Study

This study had three aims. The first aim was to examine the distribution of acculturation profiles of identification. It was expected to find the four acculturation profiles adapted from Berry’s (1997) model (integration, assimilation, separation, and marginalization) or variations thereof that only deviate slightly. It was hypothesized that the integration profile would be the largest acculturation profile (Hypothesis 1), because of the previous finding that integration is the most common acculturation profile of identification among immigrant adolescents in mid-to-late adolescence (Spiegler et al. 2019). The second aim was to characterize the development of acculturation profiles of identification. Early-to-mid adolescence is the time where most immigrant adolescents engage in intensified identity work (Erentaitė et al. 2018), exploring their social identities and sometimes re-considering alternatives (Crocetti et al. 2008). Thus, it was assumed that acculturation profiles of identification would be rather unstable, as characterized by stability probabilities within acculturation profiles of identification over time in the low to medium range (Hypothesis 2). Based on the idea that national identity is added to an already existing ethnic identity (Fleischmann and Verkuyten 2016) it was further expected that the integration profile would increase over time (Hypothesis 3). The third aim was to study the influence of social context on the development of acculturation profile of identification. Based on the idea that group boundaries are less permeable for Turkish-origin adolescents, it was expected that they would be less likely to transition from separation to integration than resettler-origin adolescents would (Hypothesis 4). To test the reactive identification idea (Verkuyten 2018) that immigrant adolescents who feel rejected by the dominant society identify more strongly with their ethnic group and start to reject the national mainstream, it was hypothesized that experiences of ethnic discrimination would predict transitions from integration to separation (Hypothesis 5).

## Method

### Participants and Procedure

Data for this study came from the on-going project (name of project and citation removed for blind review), a longitudinal study of over 2500 students in the federal state of North Rhine-Westphalia in Western Germany. Sampling followed a cohort-sequence design of the 5th–7th grades in nine lower-level secondary, intermediate secondary, and comprehensive schools^1^. To ensure a large enough sample of ethnic minority students to conduct meaningful analyses, the study targeted schools with higher shares of ethnic minority students, which was based on information on students’ citizenship provided by the federal statistics office^2^.

Data were collected in six waves in May 2013, February 2014, November 2014, September 2015, May 2016, and March 2017. Students’ participation was voluntary and written parental approval was obtained beforehand. Students filled out paper-and-pencil questionnaires in the classrooms under supervision of researchers and research assistants. In each wave, depending on the schools’ choice, either all participating students received a 5€ incentive or the respective sum was paid into the class fund. The study was conducted in two funding periods. All schools agreed to participate in the first funding phase covering three waves. For the second funding phase, six schools agreed to continue the study for three additional waves. The sample for the following analyses was restricted to five waves because the oldest year grade (i.e., those in the seventh grade at the first wave) left school after the fifth wave (i.e., after the tenth grade). Thus, employing the sixth wave would have resulted in a substantial drop in case numbers (i.e., 432 students would have been dropped).

In total, the analytical sample comprised 1076 ethnic minority students (i.e., students who were themselves born in another country (207 students), or who were born to at least one parent (732) or grandparent (126) who was born abroad)^3^. The analyses focused on 358 Turkish-origin students and 123 students from countries of the former Soviet Union, encompassing students from Russia (62.6%), Kazakhstan (25.2%), Ukraine (5.7%), Lithuania (3.3%), Belarus (1.6%) and Armenia (1.6%). Both immigrant groups were very similar in terms of their composition concerning immigrant generation and participation in the survey (see Table 1). These two groups were labeled “Turkish-origin students” and “resettler-origin students.” The mean age of students in the sample was 14.2 years (SD = 1.54; Range = 10.8–19.7) and the mean socio-economic status (captured by the highest International Socio-Economic Index of Occupational Status of students’ parents) was 32.39 (SD = 12.79; Range = 14.6–88.7), indicating the average student having a rather medium-level socio-economic background. As shown in Table 1, both groups were very similar with regard to SES.

**Table 1:** Sample characteristics

Characteristic	Turkish-origin students	Resettler-origin students
Total	358	123
Migration generation		
1	11.5%	24.8%
2	82.7%	72.7%
3	5.8%	2.5%
Sample size in wave		
1	247	89
2	273	92
3	286	94
4	276	97
5	258	99
Participation frequency		
1	9.8%	9.8%
2	9.8%	9.8%
3	15.6%	13.8%
4	26.0%	21.1%
5	38.8%	45.5%
Socio-economic status (average ISEI)	32.3	32.8
Discrimination (average)	1.67	1.51

### Measures

#### Ethnic and national identification

The measures of ethnic and national identification used items that were oriented on existing scales, such as the MEIM-R (Phinney and Ong 2007a) and the EIS (Umaña-Taylor et al. 2004), and adapted to the German context. For both ethnic and national identification, students answered seven items (“To belong to Germany/my family’s country of origin is an important part of myself.”; “I am satisfied to belong to Germany/my family’s country of origin.”; “I am glad to belong to Germany/my family’s country of origin.”; “It bothers me if somebody speaks ill about Germany/my family’s country of origin.”; “Germany/My family’s country of origin is dear to me.”; “I feel strongly attached to Germans/people from my family’s country of origin.”; “I feel like I am part of Germany/my family’s country of origin.”). The identification items were comprehensively tested in several prestudies, which included two cognitive pretests as well as tests for measurement equivalence, reliability, and construct validity (citation removed for blind review). In particular, the identification measures were shown to be invariant for different immigrant generations as well as across age groups. Students answered these questions on five-point scales ranging from “does not apply at all” over “neither nor” to “fully applies.” These seven-item scales had good psychometric properties (*α*_ethnic identification_ = 0.93, *α*_national identification_ = 0.91).

#### Auxiliary validation measures for assessing profile solutions

To validate the acculturation profiles of identification as identified by the latent profile analysis (see below), additional measures were used that should correlate differentially with specific types of profiles.

##### Ethnic-national self-categorization

This was assessed using a five-point scale indicating whether students viewed themselves “only as German” over “both to the same extent” to “only as from my family’s country of origin.”

##### Dual identification

This surveyed whether students regarded themselves as both German and a member of their family’s country of origin (on a five point scale ranging from “does not apply at all” over “neither nor” to “fully applies”).

##### Impermeability of national identity

This reflects a mean index of two items, gauging the possibility for people from their family’s country of origin to be German (“For people from my family’s country of origin it is difficult to be regarded as German by Germans.”; “For people from my family’s country of origin it is impossible to be regarded as German by Germans.”; five-point scale ranging from “completely disagree” over “neither nor” to “fully agree”; *α* = 0.76).

##### Attitudes toward ethnic majority members

This indicated the extent to which students liked Germans, and was assessed using a five-point scale ranging from “not at all” over “neither nor” to “very much.”

##### Grade point average

This shows the average of the grades in mathematics, German, and English. In the German school system, grades range from one to six with lower values indicating higher academic achievement.

##### Proportion of ethnic majority friends

This shows the average share of German ethnic majority students among the students’ friends in the grade (Students selected their ten best friends from a roster that listed all students in their grade; see [citation removed for blind review] for details).

##### Perceived ethnic discrimination

Experiences of ethnic discrimination were indexed with three items (“How often does it occur that German children or youth speak badly about you because of your family’s country of origin?”; “How often does it occur that German children or youth insulted or offended you because of your family’s country of origin?”; “How often does it occur that German children or youth treated you badly or unfairly because of your family’s country of origin?”; *α* = 0.86). Students scored their answers on a four-point scale (1 “never”, 2 “seldom”, 3 “sometimes”, and 4 “often”).

### Analytic Strategy

Latent profile analysis (LPA) and its longitudinal extension, latent transition analysis (LTA; Collins and Lanza 2010), were used in order to discover, describe, and follow qualitatively distinct groups of combinations of ethnic and national identification over time. In the first step, LPA was employed to group students into a certain number of profiles according to the extent of both their ethnic and national identification. This first step aimed to find the number of profiles with the best fit to the data, meaning that it best discriminates between the students within the space spanned by their ethnic and national identifications. This was done for each of the waves separately to test whether the LPAs would discover the same number of profiles in each wave while also considering whether the substantive meaning of profiles (e.g., the distribution of ethnic and national identification within profiles) was consistent across waves.

Models were estimated using MPlus 8.4 (Muthén and Muthén 2012–2017), using full information maximum likelihood (FIML) estimation to handle missing data (i.e., using all available data points to estimate parameters). In deciding about what “best fit” meant, a set of five criteria was used, adopted from previous research, to assess fit both in statistical and in substantive terms (Meeus et al. 2010; Nagin 2005). The first criterion stated that a model with one profile more provided a superior fit if it showed a lower value of the Bayesian information criterion (BIC). Second, following the same logic, the bootstrapped Lo–Mendel–Rubin likelihood ratio (BLRT) should be significantly lower (Nylund et al. 2007). Third, entropy should be higher than 0.70, indicating good overall classification accuracy (Reinecke 2006). Fourth, each profile should be substantively meaningful, representing a sufficient number of students in the sample. Fifth, across all five waves the substantive meaning of the profiles should be equivalent (Collins and Lanza 2010). This was inspected visually using plots that showed the attributions of students to the profiles on a two-dimensional space spanned by their ethnic and national identification. Taken together, according to these criteria, the first step identified the number of profiles for each of the two immigrant groups that were replicated in each of the five waves. Thereafter, these profiles were described to derive a substantive understanding of the meaning of these profiles.

In the second step, transition probabilities between these profiles were estimated over time using a LTA model (Kaplan 2008). This model was based on transitions between the first, third, and fifth wave. The reason for not using all five waves was infeasible computational complexity^4^. Due to the equally spaced duration between the waves of one and a half years, the overall duration between the first and last time point remains 3 years (i.e., 4 × 9 months). Again, FIML estimation was used to deal with missing data. Full measurement invariance and non-stationary transition probabilities were specified in these models. For full measurement invariance within-profile intercepts were constrained to be equal across all time points (Newsom 2015). This means that the same number and type of profiles occur at all time points and thus interpretation of transition probabilities is simple because the meaning of profiles stays constant across time (Nylund 2007). Non-stationary transition probabilities imply that transition probabilities are not the same across time points, allowing for a modeling of discontinuous change processes (e.g., more transitions between the first and third than between third and last measurement point).

In the third step, it was investigated whether perceived discrimination (modeled as a continuous covariate) at the first and third measurement point influenced the transition probabilities identified in the second step. Since FIML is not implemented in estimating transitions between the classes with regard to variation in other variables that feature missing values, missing values were imputed for discrimination. Missing values on discrimination were very low with only 5.9% of the cases, imputation was achieved by a series of carrying values backward (for wave 1) and forward (for wave 3 and 5) for all students.

## Results

The presentation of results follows the logic set forth in the analytical strategy. First, results of the LPAs are presented that show which profile solution fits the data best. Second, longitudinal transition probabilities between these profiles were estimated. Third, it was examined whether discrimination moderates transition probabilities. All three steps were carried out separately for Turkish-origin and for resettler-origin students. Basic descriptive information (*M*, SD) and correlations between study variables are displayed in Tables 2–4 for Turkish-origin, resettler-origin students, and all students with migration background, respectively.

**Table 2:** Correlations between study variables among Turkish-origin students in wave 1 (*N* = 247)

	*M*	SD	(1)	(2)	(3)	(4)	(5)	(6)	(7)	(8)	(9)
1. Ethnic-national self-categorization	3.56	1.12	1								
2. Dual identification	3.54	1.48	−0.36***	1							
3. Impermeability of national identity	3.12	1.25	0.13	0.05	1						
4. Attitudes toward ethnic majority members	4.22	0.88	−0.27**	0.31***	−0.07	1					
5. GPA	2.97	0.75	0.03	−0.05	0.17	−0.11	1				
6. Proportion of ethnic majority friends	18.33	21.12	−0.21	0.00	0.00	0.10	0.13	1			
7. Ethnic discrimination	1.62	0.74	0.08	−0.00	0.35***	−0.19	0.09	−0.10	1		
8. Ethnic identification	4.52	0.69	0.40***	0.02	0.23*	−0.09	0.09	−0.17	0.12	1	
9. National identification	3.20	1.02	−0.32***	0.30***	0.03	0.53***	−0.16	0.08	−0.12	0.02	1

All measures except for GPA and proportion of ethnic majority friends ranged from 1 to 5. Ethnic-national self-categorization was coded such that higher values indicate stronger ethnic self-categorization. Grades 1–6; higher values indicate lower achievement

*GPA* grade point average

*p < 0.05; **p < 0.01; ***p < 0.001

**Table 3:** Correlations between study variables among resettler-origin students in wave 1 (*N* = 89)

	*M*	SD	(1)	(2)	(3)	(4)	(5)	(6)	(7)	(8)	(9)
1. Ethnic-national self-categorization	2.97	1.01	1								
2. Dual identification	4.18	1.07	−0.12	1							
3. Impermeability of national identity	2.94	0.94	0.18	−0.18	1						
4. Attitudes toward ethnic majority members	4.57	0.72	−0.32	0.21	−0.13	1					
5. GPA	2.97	0.72	0.09	0.12	0.24	−0.09	1				
6. Proportion of ethnic majority friends	35.74	27.31	−0.15	−0.02	−0.03	0.20	0.01	1			
7. Ethnic discrimination	1.47	0.63	0.11	−0.13	0.14	−0.10	0.23	−0.13	1		
8. Ethnic identification	4.16	0.80	0.28	0.26	0.07	−0.14	0.22	−0.14	0.24	1	
9. National identification	3.31	0.88	−0.41***	0.11	0.05	0.49***	−0.17	0.07	0.03	0.04	1

All measures except for GPA and proportion of ethnic majority friends ranged from 1 to 5. Ethnic-national self-categorization was coded such that higher values indicate stronger ethnic self-categorization. Grades 1–6; higher values indicate lower achievement

*GPA* grade point average

***p < 0.001

**Table 4:** Correlations of study variables among students with migration background in wave 1 (*N* = 685)

	*M*	SD	(1)	(2)	(3)	(4)	(5)	(6)	(7)	(8)	(9)	(10)	(11)	(12)	(13)
1. Ethnic-national self-categorization	3.29	1.13	1												
2. Dual identification	3.79	1.41	−0.29***	1											
3. Impermeability of national identity	2.94	1.19	0.13	−0.02	1										
4. Attitudes toward ethnic majority members	4.39	0.86	−0.30***	0.28***	−0.11	1									
5. GPA	2.94	0.78	0.07	−0.05	0.12	−0.07	1								
6. Turkish ethnic origin	36.50	–	0.17**	−0.13	0.11	−0.15*	0.03	1							
7. Non-believers	16.86	–	−0.06	−0.01	−0.11	0.08	0.00	−0.13*	1						
8. Christian	40.09	–	−0.22***	0.14*	−0.05	0.14*	−0.03	−0.57***	−0.37***	1					
9. Muslim	43.05	–	0.26***	−0.13	0.14	−0.19***	0.03	0.67***	−0.39***	−0.71***	1				
10. Attitudes toward ethnic majority members	29.30	27.05	−0.22***	0.13	−0.05	0.18***	0.00	−0.30***	0.16**	0.25***	−0.37***	1			
11. Ethnic discrimination	1.62	0.78	0.09	−0.05	0.19***	−0.13	0.05	0.01	−0.05	0.01	0.03	−0.12	1		
12. Ethnic identification	4.39	3.24	0.40***	0.03	0.18***	−0.10	0.15*	0.14*	−0.16**	−0.14	0.26***	−0.23***	0.19***	1	
13. National identification	3.24	0.98	−0.33***	0.28***	0.09	0.47***	−0.11	−0.03	−0.05	0.11	−0.07	0.10	−0.03	0.05	1

Ethnic-national self-categorization was coded such that higher values indicate stronger ethnic self-categorization

*GPA* grade point average (higher values indicate lower achievement)

*p < 0.05; **p < 0.01; ***p < 0.001

### Latent Profile Analysis

#### Identifying the number of profiles

As outlined earlier, the first task was to find the number of profiles that best describes both ethnic groups’ patterns of ethnic and national identification. To this end, starting with Turkish-origin students, LPA models for two, three, and four-profile specifications were tested according to the five criteria outlined above. The overall classification accuracy was very high, indicated by an entropy measure of at least 0.91 in every wave and every model specification. Without exception, both the BIC and BLRT model selection criteria favored higher numbers of profiles, i.e., a four-profile solution. The statistical criteria therefore advocated for four profiles (see Table A1 of the Supplementary Materials). This held also true for the visual inspection of the substantive meaning of the profile attributions. As shown in Figure A1a of the Supplementary Materials, over time, the substantive meaning of each profile changed in the three-profile solution, rendering this solution unfeasible. The four-profile solution, however, although featuring only a small share of students in one profile in two waves, reflected the same profiles in substantive terms throughout all waves. As a result, in the following, four acculturation profiles of identification were distinguished among Turkish-origin students highlighting differences in their ethnic and national identification.

For resettler-origin students, the overall classification accuracy was also very high with an entropy measure of at least 0.88. Across all models, both the BIC and BLRT again favored higher numbers of profiles (see Table A2 of the Supplementary Materials). However, this did not hold for the more substantive criteria as well. As shown in Figure A1b in the Supplementary Materials, over time, the substantive meaning of each profile changed drastically in the four-profile solution, and with regard to profile sizes the three-profile solution was preferable because in the four-profile solution the absolute numbers of students attributed to the smallest profile was very low. Therefore, three acculturation profiles of identification were distinguished among resettler-origin students.

#### Describing the profiles: ethnic and national identification

**Fig. 1:**
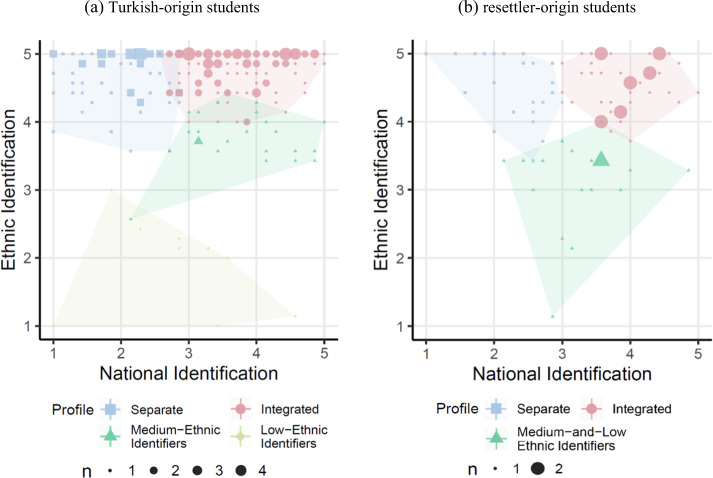
Joint distribution of ethnic and national identification across profiles at first wave of observation. **a** Turkish-origin students; **b** resettler-origin students

Figure 1 displays students’ probabilistic allocation to the acculturation profiles of identification according to (the averages of) their ethnic and national identification in the first wave. Confirming Hypothesis 1, the dominant profile (56.3% of the Turkish-origin and 49.4% of the resettler-origin students in first wave) were students that identified strongly with both their family’s country of origin (on average, 4.8 for Turkish-origin and 4.5 for resettler-origin students) and Germany (3.7 and 3.9). In line with Berry’s typology, this profile was named Integrated (red colored dots in Fig. 1). The second prominent profile in both ethnic groups (28.7 and 27.0%) featured those who identified very strongly with their family’s country of origin (4.7 and 4.5) but only weakly with Germany (2.0 and 2.3); again following Berry’s typology, this group was named Separated (blue colored squares in Fig. 1).

For Turkish-origin students two smaller profiles distinguished students primarily due to their level of ethnic identification. Members of the third profile (11.3% of Turkish-origin students in first wave) ranged at the medium-level of Turkish identification (on average, 3.8) and at medium-to-high levels of German identification (3.7), named Medium-Ethnic Identifiers (green colored triangles in Fig. 1a). The fourth profile comprised a small group of students (3.6%) who identified only very little with their Turkish heritage (1.9) and were diverse in their level of German identification, named Low-Ethnic Identifiers (yellow colored diamonds in Fig. 1a).

For resettler-origin students, the third profile (23.6%) featured students with low and medium ethnic identification, ranging average on both dimensions (on average, 3.2 on national and 3.0 on ethnic identification), named Medium- and Low-Ethnic Identifiers (green colored triangles in Fig. 1b). Overall, these results provided mixed support for interpretations of Berry’s model that equate contact or participation with other groups in society with national identification, replicating the integration and separation profiles but finding no evidence for the assimilation or marginalization profiles.

#### Describing the profiles: auxiliary validation measures

The substantive meaning of the acculturation profiles of identification was examined using basic demographics and auxiliary variables for the first wave. For Turkish-origin students, Table 5 shows that those in the Medium-ethnic identifiers profile were less likely to be female and more likely to be older. Those in the Separated profile were more likely to self-categorize as only Turkish than members of all other profiles, had the lowest scores on dual identification, had medium to high scores on perceived impermeability of national identity, the least favorable attitudes toward ethnic majority members, the lowest scores on academic achievement, and the lowest share of German friends. Students of the Integrated profile differed statistically from those of the Separated profile by self-categorizing less often as only Turkish, subscribing more often to a dual identification, having more positive attitudes towards Germans, and higher academic achievement. Furthermore, students in the two profiles Medium-Ethnic Identifiers and Low-Ethnic Identifiers were overall more similar to those in the Integrated than in the Separated profile. However, compared to the Integrated, they even less often self-categorized as only Turkish (1.07 points lower with *p* < 0.001 for Medium-Ethnic Identifiers; 1.19 points lower with *p* < 0.05 for Low-Ethnic Identifiers). The Medium-Ethnic Identifiers perceived German national identity to be most permeable. Regarding the share of German friends, Low-Ethnic Identifiers featured a substantively higher share of German students in their circle of friends compared to those students of the Separated profile. Students of the Separated profile were not only less adjusted in terms of academic achievement, they also liked Germans least and the identification measures indicated that they also perceived their Turkish identity as separate from being German.

**Table 5:** LPA descriptive means (SD) for Turkish-origin students for wave 1 (*N* = 247)

	Separated	Integrated	Medium-ethnic identifiers	Low-ethnic identifiers
Female (in %; range 0–100)	57.7^a^	51.1^a,b^	35.7^b^	44.4^a,b^
Age (range: 10.8–16.8)	12.69^a,b^ (1.21)	12.46^a^ (1.05)	12.97^b^ (1.06)	12.75^a,b^ (0.69)
Academic year (range: 5–7)	6.08^a^ (0.86)	5.90^b^ (0.84)	6.29^a,c^ (0.81)	6.33^a,b,c^ (0.50)
Self-categorization as Turkish (range 1–5)	4.12^a^ (0.86)	3.57^b^ (1.05)	2.50^c^ (0.96)	2.38^c^ (1.41)
Dual identification (range 1–5)	2.97^a^ (1.52)	3.83^b^ (1.42)	3.74^b,c^ (1.35)	3.11^a,b^ (1.36)
Impermeability (range 1–5)	3.21^a^ (1.30)	3.26^a^ (1.15)	2.48^b^ (1.16)	2.44^a,b^ (1.45)
Liking Germans (range 1–5)	3.67^a^ (1.07)	4.41^b^ (0.68)	4.46b^b^ (0.74)	4.57^b^ (0.79)
GPA (range 1–6)	3.11^a^ (0.68)	2.90^b^ (0.77)	2.93^a,b^ (0.79)	3.03^a,b^ (0.75)
German friends (in %; range 0–100)	16.4^a^ (22.2)	17.6^a,b^ (19.2)	21.5^a,b^ (24.8)	33.7^b^ (25.6)

Means that do not share superscripts are significantly different at *p* < 0.05

For resettler-origin students, Table 6 shows that the composition of the three profiles was more similar than among Turkish-origin youth. There were no significant differences between profiles in basic demographics. It was echoed that students of the Separated class most often self-categorized as members their family’s country of origin and liked Germans the least compared to students of the other two profiles. Overall, these helped to validate the identified profile solutions and suggested that these differ in meaningful ways from one another as one would expect.

**Table 6:** LPA descriptive means (SD) for resettler-origin students for wave 1 (*N* = 89)

	Separated	Integrated	Low- and medium-ethnic identifiers
Female (in %; range 0–100)	62.5	52.3	52.4
Age (range: 10.8–14.6)	12.88 (0.98)	12.56 (1.06)	12.75 (0.89)
Academic year (range 5–7)	6.33 (0.76)	5.98 (0.90)	6.29 (0.78)
Self-categorization as one from my family’s country of origin (range 1–5)	3.61^a^ (1.12)	2.77^b^ (0.90)	2.65^b^ (0.81)
Dual identification (range 1–5)	4.17^a,c^ (1.27)	4.38^a,b^ (0.94)	3.72^a,c^ (1.02)
Impermeability (range 1–5)	2.82 (0.95)	3.06 (1.02)	2.84 (0.73)
Liking Germans (range 1–5)	4.17^a^ (0.89)	4.72^b^ (0.59)	4.70^b^ (0.57)
GPA (range 1–6)	3.12 (0.70)	2.91 (0.68)	2.90 (0.85)
German friends (in %; range 0–100)	37.9 (27.6)	33.7 (29.0)	37.6 (24.3)

Means that do not share superscripts are significantly different at *p* < 0.05

### Latent Transition Analysis

#### Changes in profile sizes over time

**Fig. 2:**
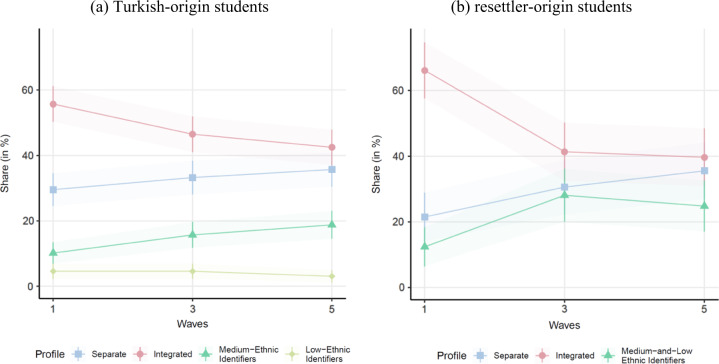
Profile shares across the observation period. **a** Turkish-origin students; **b** resettler-origin students

Going beyond the mere attribution of students to profiles, the LPA was extended longitudinally to an LTA to examine to what extent students’ attributions to the acculturation profiles of identification changed over the observation period. Figure 2 shows the time trends of profile sizes in terms of shares among all students of either Turkish- or resettler-origin. The most sizable acculturation profile of identification in the first wave, the Integrated profile, featuring around 60% of the students in both ethnic groups, decreased considerably over time to around 40% at the last observation. This finding contradicts Hypothesis 3, which expected an increase in the frequency of this profile. Furthermore, for Turkish-origin students, while the size of the Low-Ethnic Identifiers profile remained constant over time around 5%, the Separated profile increased by around 5% (from 30 to 35%) and the profile of Medium-Ethnic Identifiers doubled from around 10% to almost 20%. For resettler-origin students, both the Separated and the Medium- and Low-Ethnic Identifiers profiles almost doubled from around 20% to around 35% and from just above 10% to over 20%, respectively, at the last observation. Taken together, over time, students of both ethnic groups were less likely to be attached to the Integrated profile. For Turkish-origin students, e this trend was accompanied by an increase in the size of profiles featuring less ethnic identification. For resettler-origin students this trend was accompanied primarily with an increase in profiles featuring less national identification.

#### Transitions between acculturation profiles of identification over time

Having described the overall patterns of acculturation profile sizes over time, it was further investigated to what extent individual students stayed in the same profile, to what extent their profile attribution changed over time, and, if the latter applied, from which origin to which destination profile they transitioned. Partially supporting Hypothesis 2, for both ethnic groups there was considerable change of profile attributions over time with stability coefficients in the medium range^5^ (*M*_stability_ = 0.58; SD = 0.09).

Starting with Turkish-origin students, Table 7 displays the transition probabilities between the four profiles across the three time points. For students in the Separated profile, over time stability was 0.58 and 0.70 for both transition periods, respectively, suggesting that most students in this group did not change and that stability increased over time. For those students who did change, the most likely transition was to the *integrated* profile, with transition probabilities decreasing from 0.34 to 0.23 across both transition periods. In the Integrated profile, over time stability was also medium (0.58–0.60) but did not increase. At the first transition, students in this profile were more likely to transition to the Separated (0.25) than to the Medium-Ethnic Identifiers Profile (0.14). However, at the second transition, they were equally likely to transition to either Separated (0.18) or Medium-Ethnic Identifiers (0.19). Among Medium-Ethnic Identifiers over time stability was also medium, ranging from 0.41 to 0.46, respectively. When students transitioned, they were most likely to transition to the Integrated profile (0.28–0.34); at the first transition period they were also likely to transition to Separated (0.24), but at the second observation period this option became more unlikely (0.10). Among the smallest group of low-ethnic identifiers, over time stability was also moderate (0.53–0.46) and there were no clear patterns of transitions to particular profiles.

**Table 7:** Transition probabilities for Turkish-origin students (*N* = 344)

	Wave 3				Wave 5			
	Separated (33.23%)	Integrated (46.46%)	Medium-ethnic identifiers (15.69%)	Low-ethnic identifiers (4.62%)	Separated (35.69%)	Integrated (42.46%)	Medium-ethnic identifiers (18.77%)	Low-ethnic identifiers (3.08%)
Wave 1								
Separated (29.5%)	0.58	0.34	0.06	0.02	0.70	0.23	0.08	0.00
Integrated (55.69%)	0.25	0.58	0.14	0.03	0.18	0.60	0.19	0.03
Medium-ethnic identifiers (10.15%)	0.24	0.28	0.41	0.07	0.10	0.34	0.46	0.10
Low-ethnic identifiers (4.61%)	0.21	0.11	0.15	0.53	0.13	0.09	0.33	0.46

Table 8 displays the transition probabilities for resettler-origin students. Among students in the Separated profile, over time stability was moderately high, ranging from 0.65 to 0.63. At the first transition, students in this profile who did move were equally likely to transition to either Integrated (0.18) or Low-and Medium Ethnic Identifiers (0.17). In the second transition period, they were slightly more likely to transition to Low and Medium Ethnic Identifiers (0.23) than to the Integrated profile (0.14). The lower probabilities to transition from Separated to Integrated compared to Turkish-origin students disconfirm the differential group permeability hypothesis (Hypothesis 4). Among students in the Integrated profile, over time stability increased from 0.55 to 0.70. In the first transition period, they were equally likely to transition to either Separated (0.21) or Low- and Medium-Ethnic Identifiers (0.24). However, at the second transition period, they were more likely to transition to Separated (0.25) than to Low- and Medium-Ethnic Identifiers (0.04). Among Low- and Medium-Ethnic Identifiers over time stability was also moderately high (0.65–0.62). There were no clear patterns of transitions to particular profiles among students in this profile as students were equally likely to transition to either Separated (0.19–0.23) or Integrated (0.17–0.15).

**Table 8:** Transition probabilities for resettler-origin students (*N* = 121)

	Wave 3			Wave 5		
	Separated (30.56%)	Integrated (41.32%)	Low- and medium-ethnic identifiers (28.10%)	Separated (35.54%)	Integrated (39.67%)	Low- and medium-ethnic identifiers (24.79%)
Wave 1						
Separated (21.48%)	0.65	0.18	0.17	0.63	0.14	0.23
Integrated (66.12%)	0.21	0.55	0.24	0.25	0.70	0.04
Low- and medium-ethnic identifiers (12.40)	0.19	0.17	0.65	0.23	0.15	0.62

#### Transitions moderated by perceived discrimination

In order to test the reactive identification hypothesis (Hypothesis 5), it was tested whether these transition probabilities were moderated by perceived discrimination. The latent transition analysis with covariates was specified with lagged effects such that, e.g., perceived discrimination in wave 1 was related to class changes between waves 1 and 3 and perceived discrimination in wave 3 to transitions from wave 3 to 5. Because sample size was relatively low in both ethnic minority groups the inclusion of this covariate led to combinations of classes, which had few observations, causing zero variance in the covariate. This meant that some slopes could not be estimated. Therefore, only the results with the full sample of ethnic minority students (*n* = 1016) are reported, including Turkish-origin and resettler-origin students and all other ethnic minority students. This analysis suggested that perceived discrimination affected classification probabilities, but not transition probabilities. That is, ethnic minority students who reported to be discriminated more often were less likely to be in the medium- and low ethnic identifier profile at wave 1 (OR = 0.65, SE = 0.12, *p* = 0.003) than in the integrated profile. However, there was no significant effect of perceived discrimination on the transition probabilities across profiles over time (all *p*s > 0.05; see Table B4 of the Supplemental Materials). This suggests that while perceived discrimination decreased the likelihood for ethnic minority students to be in the medium-and low ethnic identifier profile at wave 1, it did not affect whether students stayed in or moved between particular profiles. Thus, results did not support the reactive identification hypothesis (Hypothesis 5).

### Sensitivity Analyses

As a robustness check LPAs and LTAs (without covariates) were repeated with the full sample of ethnic minority students (*n* = 1016), including Turkish-origin and resettler-origin students and all other ethnic minority students. These results can be found in the Supplementary Materials (Part B). Results pointed to a three-class solution that was similar in content to the solution found among resettler-origin students. All other results were similar to the analyses presented above. The classes were distinct from each other as shown by differential means on the auxiliary validation variables. Transition stabilities were in the medium range with most transitions occurring between the separated and integrated classes. Integration decreased substantially over the course of the study.

## Discussion

Ethnic minority adolescents are tasked to find a balance between their ethnic and national identities, resulting in different acculturation profiles of identification. While ample research has documented links between particular acculturation profiles (e.g., integration) and psychological adjustment, little is known about how these profiles develop—that is, how ethnic minority adolescents come to adopt a certain acculturation profile, how stable these profiles are over time, and what may predict transitions between profiles. Therefore, this study used a person-centered approach to examine the distribution and development of acculturation profiles of identification among a sample of Turkish-origin and resettler-origin adolescents in Germany.

### Distribution and Meaning of Acculturation Profiles of Identification

Overall, results from the latent profile analyses provided mixed support for Berry’s model, replicating the integration and separation profiles in both ethnic minority groups but finding no evidence for the assimilation or marginalization profiles in either of them. In line with Hypothesis 1, the Integrated profile was the largest profile in both groups, comprising well over half of the sample. Previous research has found support for the separation, assimilation, and integration profiles while marginalization was not always found and if so it concerned usually a very small group (Schwartz and Zamboanga 2008). So, it is more surprising that results provided no evidence for assimilation. However, it is important to note that this study focused only on the identity domain of acculturation and did not assess other domains, such as cultural practices or values. Another reason why no evidence was found for assimilation may have to do with the examined immigrant groups and the context of the study. Turkish-origin youth tend to identify very strongly with their ethnic group (Verkuyten 2018) and accordingly the profile of low ethnic identifiers was very small (4.5%) among this group. While one previous study found evidence for a growth trajectory that could be labeled as assimilation among resettler-origin youth in Germany (Stoessel et al. 2014), other research showed that immigrant students were more likely to describe themselves in terms of ethnic and overlapping self-views while only a small minority (5.8%) had a purely German self-view (Hannover et al. 2013). Whether ethnic minority adolescents choose assimilation may also depend on levels of ethnic diversity in the schools they are attending. In more homogeneous contexts, dominated by a large majority of non-immigrant students, ethnic minority adolescents may face higher pressures for assimilation. The examined schools, by contrast, were ethnically heterogeneous and thus youth may have felt less pressured to assimilate—in line with the balance of power principle (Juvonen et al. 2006) and previous research on the role of school ethnic composition for minority youth’ sense of national belonging (Gharaei et al. 2018).

The latent profile solutions were validated with the help of auxiliary variables. These analyses showed that across both immigrant groups, students in the Separated class were most likely to self-categorize as only a member of their ethnic/immigrant group and least likely to like Germans. In addition, among Turkish-origin students, separation was associated with significantly lower academic achievement than integration, mirroring previous research that established a positive link between integration and various indicators of sociocultural adaptation (Nguyen and Benet-Martinez 2013). Among Turkish-origin youth, Medium-Ethnic Identifiers differed from the Integrated group in that they were less likely to self-categorize only as Turkish and perceived German national identity as more permeable. Overall, these results helped to cross-validate the latent profile solutions by showing that these are, in fact, qualitatively distinct groups that differ in meaningful ways.

### Transitions between Classes over Time and Changes in Class Sizes

It was expected that due to intensified identity work during early-to-mid adolescence (Erentaitė et al. 2018) over time stability of acculturation profiles of identification would be in the low to medium range. Hypothesis 2 was only partly supported because none of the stability coefficients was low—they were all in the medium range (i.e., between 40 and 70% stability). Nevertheless, these results suggest that acculturation profiles of identification are far from stable in this developmental period. The results differ markedly from Lee et al. (2018) who found very high (0.80–1) over-time stability of identification profile solutions in a sample of Hispanic adolescents in the United States. While the sample comprised students in early to mid-adolescence, their study focused on mid-adolescence. This may suggest that acculturation profiles of identification become more stable from mid-adolescence onwards when adolescents may already have explored their identities and are more likely to have reached the stage of identity resolution (Umaña-Taylor et al. 2014). However, results of these two studies are difficult to compare for two reasons. First, different groups were compared in different countries. Second, this study found three to four profiles, depending on ethnic minority group, while Lee et al. (2018) found only two profiles. Thus, lower stability coefficients in this study could also be the result of having extracted more profiles. However, the identified latent profile analyses clearly spoke against extracting fewer profiles in this sample.

One of the most striking findings was that in direct contradiction to Hypothesis 3 the Integrated profile did not increase over time but decreased considerably among both immigrant groups over the course of the study. This hypothesis was based on the idea that national identity would be added to an already existing ethnic identity (Fleischmann and Verkuyten 2016) in the course of identity development. Results suggested, in contrast, that the Integrated profile was very large to begin with but decreased by 15% points among Turkish-origin and by 25% points among resettler-origin youth. This decline of integration is surprising given that other studies found that both Muslim ethnic minority adolescents (Spiegler et al. 2019) and resettler-origin adolescents (Stoessel et al. 2014) develop some form of dual identity over time. Except for the Separated profile, this also applied to the identified profile solutions of this study. But adding to previous research, it shows that there is more than one kind of dual identity. Results indicate that there are transitions between these profiles, which do not suggest that all ethnic minority students will end up with being high on both even though there may be increases in the underlying dimensions (i.e., ethnic and national identification). It is important to keep in mind that this study used a different analytical approach thereby revealing something previous studies could not show. These previous studies focused on increases or decreases of dual identification but could not discern whether adolescents transition between qualitatively different profiles.

To be sure, despite its decline, the Integrated profile was still the largest profile across all measurement occasions. But the results suggest that from early to middle adolescence it becomes more rather than less difficult to uphold a dual identity for ethnic minority adolescents. One reason for this may be that it is particularly the period of early-to mid-adolescence when ethnic minority youth begin to become aware of ethnic status differences in society and how their ethnic group is esteemed by others (Umaña-Taylor et al. 2014). Thus, they may start out thinking it is very easy to identify both with their ethnic and the national group. But, as much research shows, ethnic minority individuals are almost constantly challenged in regard to both their ethnic and national identities. On the one hand, ethnic majority individuals favor immigrants to assimilate and thus to disregard their ethnic heritage (Zick et al. 2001). On the other hand, when ethnic minority individuals do claim national identity this is also questioned (e.g., “where are you really from?”; Cheryan and Monin 2005). Moreover, non-immigrants in European receiving societies tend to have a unidimensional approach to immigrant acculturation, such that they assume minorities to refrain from adopting the host culture if they are presented as maintaining their origin culture, and vice versa (Van Acker and Vanbeselaere 2011). An identification pattern that combines a high sense of belonging to both groups might therefore be particularly challenging to be validated in these societal contexts, leading youth to resort to identity options that more clearly signal allegiance to a single group. To understand better whether integration occurs through adding national identity to an existing identity, future work should include younger participants (e.g., pre-adolescents). But so far, the results of this study suggest that among descendants of immigrants the development of these identities occurs in parallel rather than consecutively.

### How Do Group Permeability and Experiences of Discrimination affect Transition Probabilities?

It was assumed that national group boundaries are more permeable for resettler-origin adolescents than for Turkish-origin adolescents and therefore the former would be more likely to transition from Separated to Integrated than the latter. Again, findings disconfirmed Hypothesis 4 because resettler-origin students were less likely than Turkish-origin students to make that transition. In contrast, resettler-origin students were more likely to transition to the Medium- and Low-Ethnic Identifiers class. Thus, the assumption that those national group boundaries are more permeable for resettler-origin youth than for Turkish-origin youth may not hold. There is research to suggest that resettler-origin individuals expected to be treated and accepted as co-nationals before migrating to Germany (Hess 2016). In reality, they still face discrimination in German society (e.g., being labeled as “Russians”), resettler-origin youth are often associated with delinquency in public discourse (Titzmann et al. 2014), and their parents often struggle to get foreign degrees recognized and thus have to work in occupations below their levels of qualification (Haberfeld et al. 2011). Psychologically, expecting to be treated equally and then experiencing unequal treatment may be more difficult to cope with than not having such high expectations. This is supported by research on the Integration Paradox, which suggests that economically more integrated and highly educated immigrants turn away from national identity because of frustrated expectations (Verkuyten 2016). Though speculative, this may explain why the Integrated class decreased even more among the resettler-origin as compared to the Turkish-origin students.

Finally, further analyses tested Hypothesis 5 (Verkuyten 2018) that immigrant adolescents who perceive ethnic discrimination would start to dis-identify with the nation and identify more strongly with their ethnic group. It was thus hypothesized that ethnic minority adolescents who reported being discriminated often would be more likely to transition from integrated to separated. Findings did not support this hypothesis because discrimination had no effect on transition probabilities in the combined sample of ethnic minority students. This finding contrasts with other work that has suggested that ethnic discrimination leads to decreases in national identification in Germany (Fleischmann et al. 2019) and Finland (Jasinskaja‐Lahti et al. 2009). However, the effects of ethnic discrimination on national identification in the German study were small and both studies did not support the assumption that ethnic discrimination boosts ethnic identification. Thus, while ethnic discrimination may decrease national identification among ethnic minority adolescents this effect may not be strong enough to lead to categorical changes in profile membership. Future studies should examine group-based discrimination in addition to personal experiences of discrimination because recent research suggests that awareness that fellow ethnic group members are discriminated against has detrimental effects on psychological adjustment above and beyond personal discrimination (Stevens and Thijs 2018).

Findings showed that ethnic minority students who perceived to be discriminated against were more likely to be in the Integrated than in the Medium- and Low-Ethnic Identifiers profile at wave 1. This finding contradicts the idea that discrimination pushes individuals away from integration. A possible explanation for this finding is given by work on social identity performance (Klein et al. 2007). This work suggests that individuals may express social identities strategically in order to (re-)affirm group identities. This would suggest a positive feedback loop by which those with an integrated profile respond to discrimination by further strengthening their expressed dual identification instead of withdrawing to their ethnic group.

### Limitations and Directions for Future Research

It was not possible to disentangle chronological age from the time course of the study in the analyses. The reason for this was the study’s cohort-sequential design, which initially observed students in grades 5, 6, or 7 and then repeatedly over time. While this meant that all students grew older to the same extent, their ages differed at specific times of observation due to these initial differences in age and cohorts were not large enough to analyze them separately. Thus, while the analyses are developmental in nature, it was not possible to analyze the data in such a manner that it would allow us to draw conclusions about the development of acculturation profiles across chronological age. The latter would have allowed to examine the relative influence of discrimination on the development of acculturation profiles at different developmental periods. Future research should either use a different sampling design or larger samples to allow for such an analysis. Another issue with person-centered analyses in general is that they depend to a larger degree on specificities of the sample than variable-based approaches (Collins and Lanza 2010). Thus, future research needs to cross-validate these results with other samples, also from less ethnically heterogeneous schools. Another limitation is the moderate sample size, particularly among the resettler-origin youth. While analyses for the full sample of ethnic minority students are presented in the supplemental materials, it was a deliberate choice to do analyses on specific immigrant groups because of group-specific hypotheses. Lumping all immigrant students together creates a lot of noise in the analysis because different immigrant groups (e.g., visible vs. invisible ethnic minority) may have very different social status positions in society. Another limitation is that it was not possible to examine the *process* of identity development in terms of identity exploration and resolution, which would perhaps allow to better understand when and why certain transitions between acculturation profiles of identification occur. Future research should thus include relevant measures of ethnic and national identity exploration and resolution (cf. Umaña-Taylor et al. 2019) to examine the processes by which adolescents come to adopt a particular acculturation profile of identification.

## Conclusion

How ethnic minority adolescents combine their ethnic and national identifications has important implications for their social-emotional adjustment but to date little is known on how they develop particular acculturation profiles of identification. This study sought to characterize the development of acculturation profiles of identification among ethnic minority adolescents of Turkish and resettler-origin in Germany—one low and one high status immigrant group. The study focused on the developmental period of early-to-mid adolescence when adolescents engage in intensified identity work and become aware of status differences between ethnic groups in society. It also investigated the impact of ethnic group boundaries and ethnic discrimination. Results showed that while integration was common, acculturation profiles were volatile and integration decreased substantially over time. Results provided no evidence for greater permeability of the national category for resettler-origin students and no evidence that perceived discrimination drives transitions from integration to separation. These findings have at least three implications. First, the high volatility of acculturation profiles of identification over time suggests that a snapshot approach to studying acculturation in this developmental period is problematic. Second, in contrast to studies with older adolescents that show an increase of integration, these findings suggest that during this developmental period it becomes more difficult to maintain a dual identity. It is thus an open question whether integration rebounds in later adolescence and for whom. Third, based on these findings it would be premature to assume that integration is easier for resettler-origin adolescents than for students of Turkish-origin because of the former group’s higher status position in society. Finally, perceptions of discrimination were not responsible for categorical shifts from integration to separation, adding to other findings from Europe that have failed to support the reactive identification idea.

## Supplementary Material

Supplementary Materials

## Footnotes

1 In the German school system, students are separated after primary school (grade 1–4) into different tracks of secondary schools, depending on academic ability. The lower-level secondary track (Hauptschule) prepares for vocational schools and apprenticeships while the intermediate secondary track (Realschule) prepares for vocational schools and higher vocational schools. The comprehensive track (Gesamtschule) combines both the lower-level and intermediate track in one school. Only completion of the highest track (Gymnasium) enables students to later study at universities. Because students of immigrant origin are underrepresented at this type of schools (Kalter and Granato 2007) no data were collected in the highest track.

2 The nine participating schools belonged to three strata with different ethnic compositions (citation removed for blind review). For each stratum, one lower secondary school, one intermediate secondary school, and one comprehensive school was sampled. Based on federal registers, the strata were defined as: (1) 10–15% of students hold Turkish citizenship; (2) more than 15% of students hold Turkish citizenship; (3) <10% of students hold non-German citizenship and <5% of students hold Turkish citizenship.

3 In more detail, 686 participated in the first, 784 in the second, 823 in the third, 819 in the fourth, and 772 in the fifth wave. With regard to participation frequency, 36.5% of the students participated five times, 24.7% four times, 14.5% three times, 11.7% two times, and 12.6% once. The sample featured 77 immigrant countries, the most salient being Turkey (33.3% of the students in the sample), Poland (9.3%), the Russian Federation (7.2%), Italy (6.4%), and Kazakhstan (2.9%).

4 Specifying latent transition models, every additional time point exponentially increases computation time. Whereas a model using three waves and four classes already estimates 4^3^ = 64 transitions, a model using five waves estimates 4^5^ = 1024 transitions, which becomes computationally extremely demanding.

5 Coefficients were classified to be low (<0.3), medium (around 0.5) or high (>0.7).
