# Supplementary material for: Changes in Turkish- and Resettler-origin Adolescents’ Acculturation Profiles of Identification: A Three-year Longitudinal Study from Germany
Source: J Youth Adolesc. 2020 May 13;49(12):2476–94. doi: 10.1007/s10964-020-01250-w (PMC7585569; doi:10.1007/s10964-020-01250-w)

**Supplementary Materials**

Table of Contents

[Part A 2](#_Toc37837003)

[Table A1. Latent Profile Analysis for Turkish-origin students 2](#_Toc37837004)

[Table A2. Latent Profile Analysis for resettler-origin students 3](#_Toc37837005)

[Figure A1. Ethnic and National Identification across Profiles at all Waves of Observation 4](#_Toc37837006)

[A. Turkish-origin students 4](#_Toc37837007)

[B. Resettler-origin students 5](#_Toc37837008)

[Figure A2. Profile Shares Across Different Ages 6](#_Toc37837009)

[Part B 7](#_Toc37837010)

[Table B1. Latent Class Analysis Model Selection for All Five Waves for the Full Sample of Students with Migration Background 7](#_Toc37837011)

[Table B2. LCA Descriptive Means for Students with Migration Background for First Wave (N = 685) 8](#_Toc37837012)

[Table B3. Transition Probabilities for Students with Migration Background (N = 1,023) 9](#_Toc37837013)

[Table B4. Transition Probabilities Moderated by Discrimination for Students with Migration Background (N = 1,016) 10](#_Toc37837014)

[Figure B1. Ethnic and National Identification across Profiles at all Waves for the Full Sample of Students with Migration Background 11](#_Toc37837015)

[Figure B2. Profile Shares across the Observation Period 12](#_Toc37837016)

[Figure B3. Profile Shares Across Different Ages 13](#_Toc37837017)

# Part A

## Table A1. Latent Profile Analysis for Turkish-origin students

| Classes | Goodness of Fit | | | Size (Share in %) | | | |
| --- | --- | --- | --- | --- | --- | --- | --- |
|  | BIC | BLRT^a^ | Entropy | Class 1 | Class 2 | Class 3 | Class 4 |
| *Wave 1* (N = 247) | | | | | | | |
| 2 | 9496.6 | 727.6 | 0.98 | 8.5 | 91.5 |  |  |
| 3 | 8977.9 | 601.4 | 0.92 | 4.0 | 56.3 | 39.7 |  |
| 4 | 8774.5 | 286.0 | 0.93 | 3.6 | 11.3 | 28.7 | 56.3 |
| *Wave 2* (N = 273) | | | | | | | |
| 2 | 10309.4 | 824.4 | 0.91 | 36.6 | 63.4 |  |  |
| 3 | 9762.8 | 630.8 | 0.94 | 8.8 | 34.1 | 57.1 |  |
| 4 | 9531.1 | 315.8 | 0.92 | 6.2 | 33.0 | 41.8 | 19.0 |
| *Wave 3* (N = 286) | | | | | | | |
| 2 | 10752.8 | 1168.3 | 0.98 | 9.8 | 90.2 |  |  |
| 3 | 10105.3 | 732.4 | 0.92 | 32.9 | 59.1 | 8.0 |  |
| 4 | 9746.6 | 443.5 | 0.94 | 29.7 | 2.8 | 16.1 | 51.4 |
| *Wave 4* (N = 276) | | | | | | | |
| 2 | 10103.1 | 1277.3 | 0.97 | 16.7 | 83.3 |  |  |
| 3 | 9668.8 | 512.6 | 0.91 | 14.1 | 30.8 | 55.1 |  |
| 4 | 9296.7 | 451.1 | 0.92 | 18.5 | 29.0 | 5.1 | 47.5 |
| *Wave 5* (N = 256) | | | | | | | |
| 2 | 9481.8 | 1173.8 | 0.98 | 13.3 | 86.7 |  |  |
| 3 | 8889.7 | 674.2 | 0.98 | 6.6 | 24.2 | 69.1 |  |
| 4 | 8476.9 | 496.0 | 0.94 | 5.1 | 33.2 | 20.7 | 41.0 |
| *Note*: ^a^ Differences between the BLRT of a model with one more class versus one fewer class were significant at *p* < .001 for all waves and all 15 model comparisons. | | | | | | | |

## Table A2. Latent Profile Analysis for resettler-origin students

| Classes | Goodness of Fit | | | Size (Share in %) | | | |
| --- | --- | --- | --- | --- | --- | --- | --- |
|  | BIC | BLRT^a^ | Entropy | Class 1 | Class 2 | Class 3 | Class 4 |
| *Wave 1* (N = 89) | | | | | | | |
| 2 | 3628.9 | 216.6 | 0.93 | 22.5 | 77.5 |  |  |
| 3 | 3500.8 | 195.5 | 0.94 | 27.0 | 49.4 | 23.6 |  |
| 4 | 3468.3 | 99.8 | 0.96 | 3.4 | 24.7 | 21.3 | 50.6 |
| *Wave 2* (N = 92) | | | | | | | |
| 2 | 3528.8 | 307.9 | 0.91 | 44.6 | 55.4 |  |  |
| 3 | 3416.5 | 180.2 | 0.93 | 35.9 | 31.5 | 32.6 |  |
| 4 | 3367.2 | 116.8 | 0.95 | 31.5 | 13.0 | 18.5 | 37.0 |
| *Wave 3* (N = 94) | | | | | | | |
| 2 | 3769.1 | 343.3 | 0.96 | 33.0 | 67.0 |  |  |
| 3 | 3657.8 | 179.5 | 0.96 | 13.8 | 67.0 | 19.1 |  |
| 4 | 3579.9 | 146.0 | 0.93 | 19.1 | 11.7 | 35.1 | 34.1 |
| *Wave 4* (N = 97) | | | | | | | |
| 2 | 3914.7 | 302.0 | 0.88 | 32.0 | 68.0 |  |  |
| 3 | 3760.5 | 222.8 | 0.93 | 35.1 | 25.8 | 39.2 |  |
| 4 | 3648.3 | 180.8 | 0.94 | 4.1 | 42.3 | 24.7 | 28.9 |
| *Wave 5* (N = 99) | | | | | | | |
| 2 | 3797.3 | 324.9 | 0.90 | 38.4 | 61.6 |  |  |
| 3 | 3629.5 | 236.7 | 0.91 | 20.2 | 36.4 | 43.4 |  |
| 4 | 3532.0 | 166.4 | 0.93 | 28.3 | 32.3 | 7.1 | 32.3 |
| *Note*: ^a^ Besides comparison between four versus three classes in the fourth wave, differences between the BLRT of a model with one more class versus one fewer class were significant at *p* < .001 for the remaining 14 model comparisons. | | | | | | | |

## Figure A1. Ethnic and National Identification across Profiles at all Waves of Observation

### A. Turkish-origin students


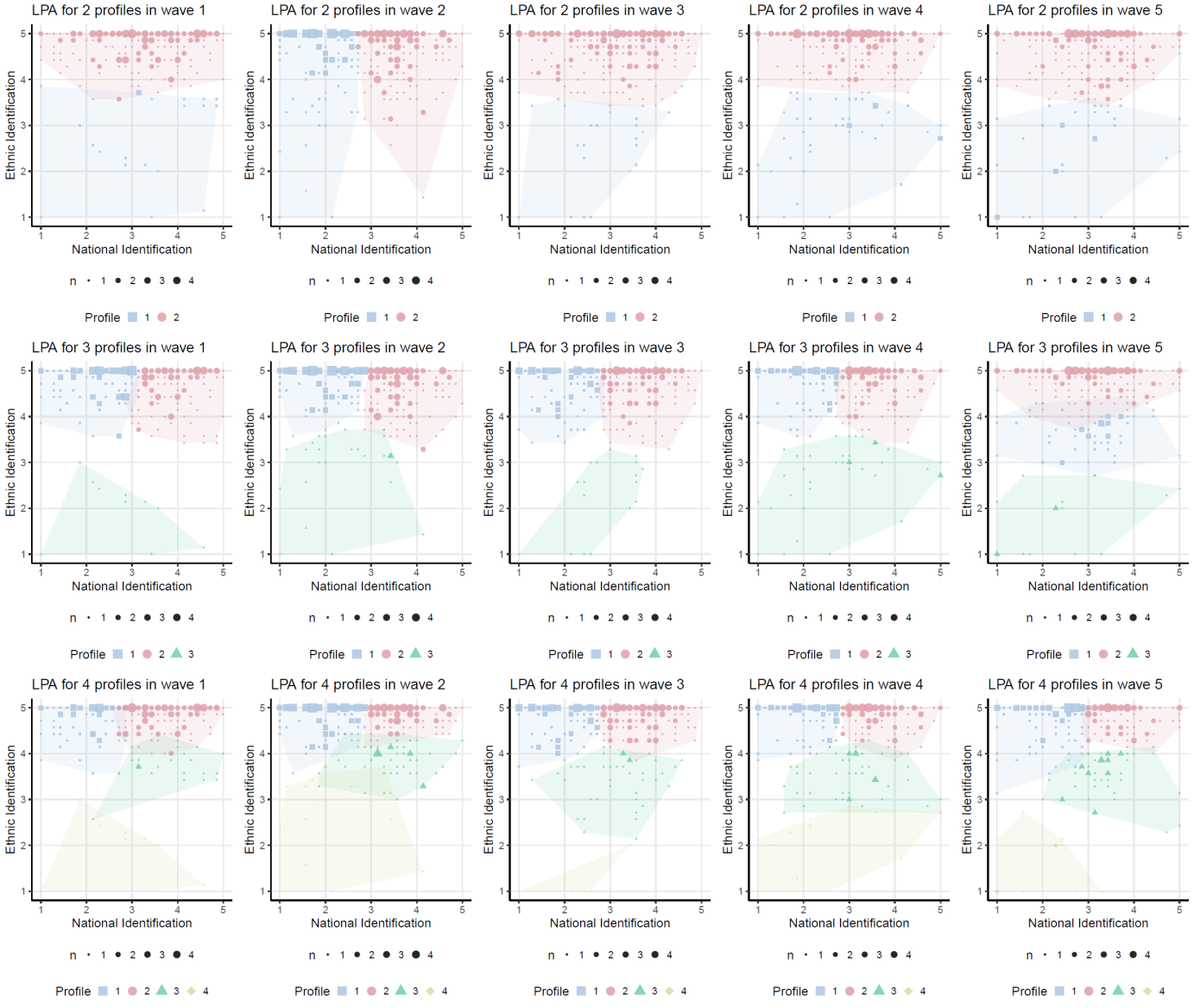


### B. Resettler-origin students


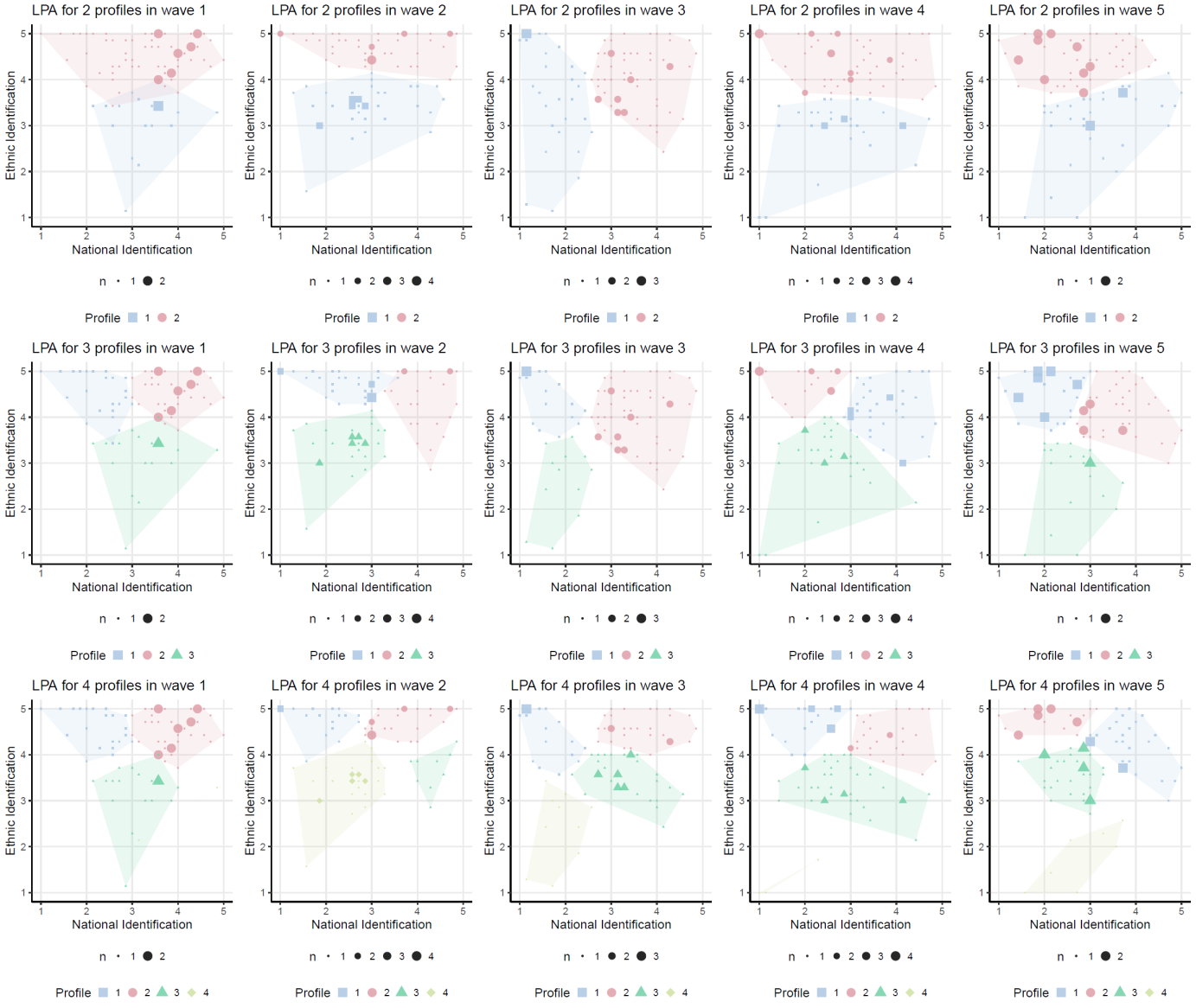


## Figure A2. Profile Shares Across Different Ages

| (a) Turkish students | (b) Resettler-origin students |
| --- | --- |
| 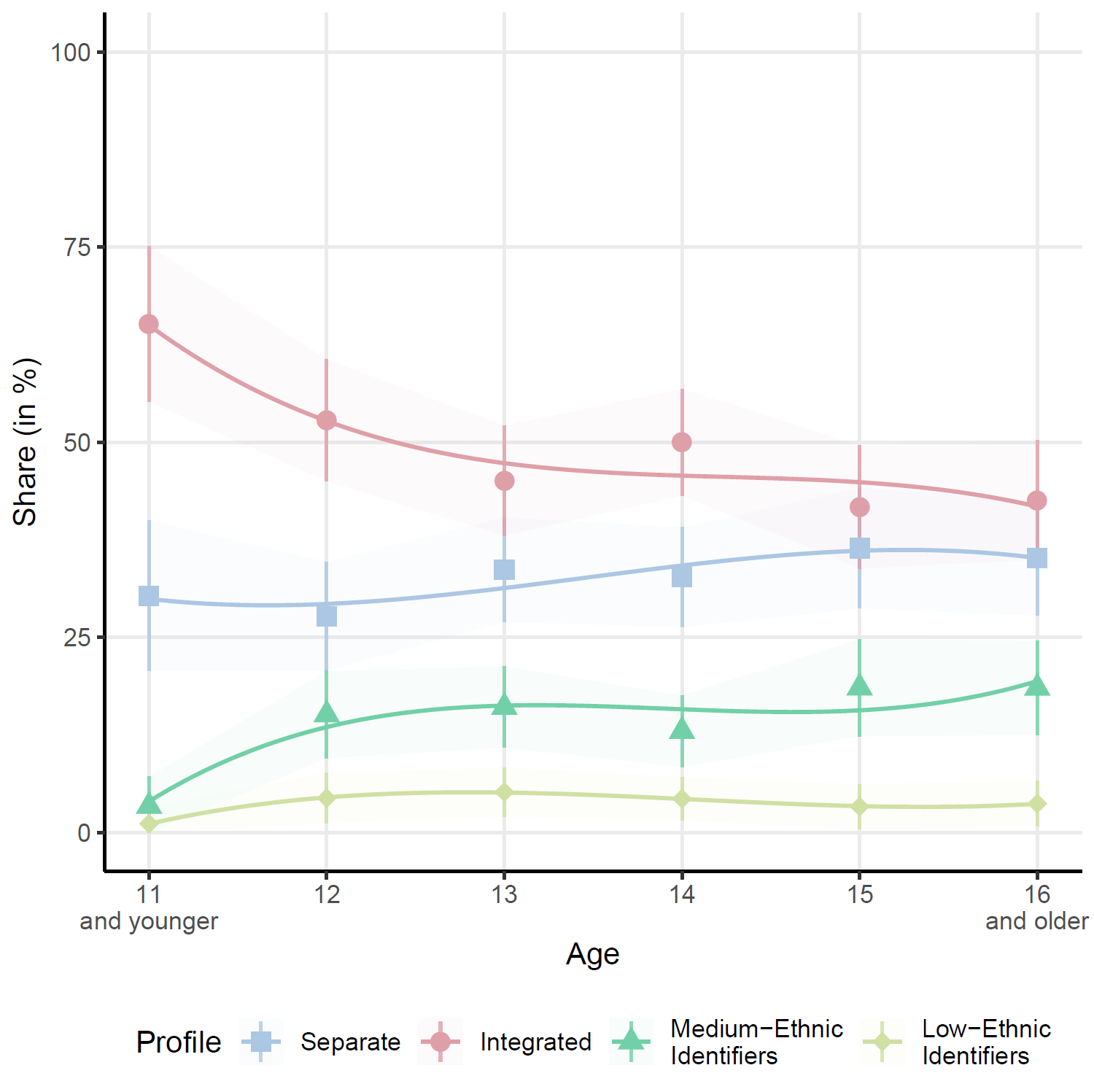 | 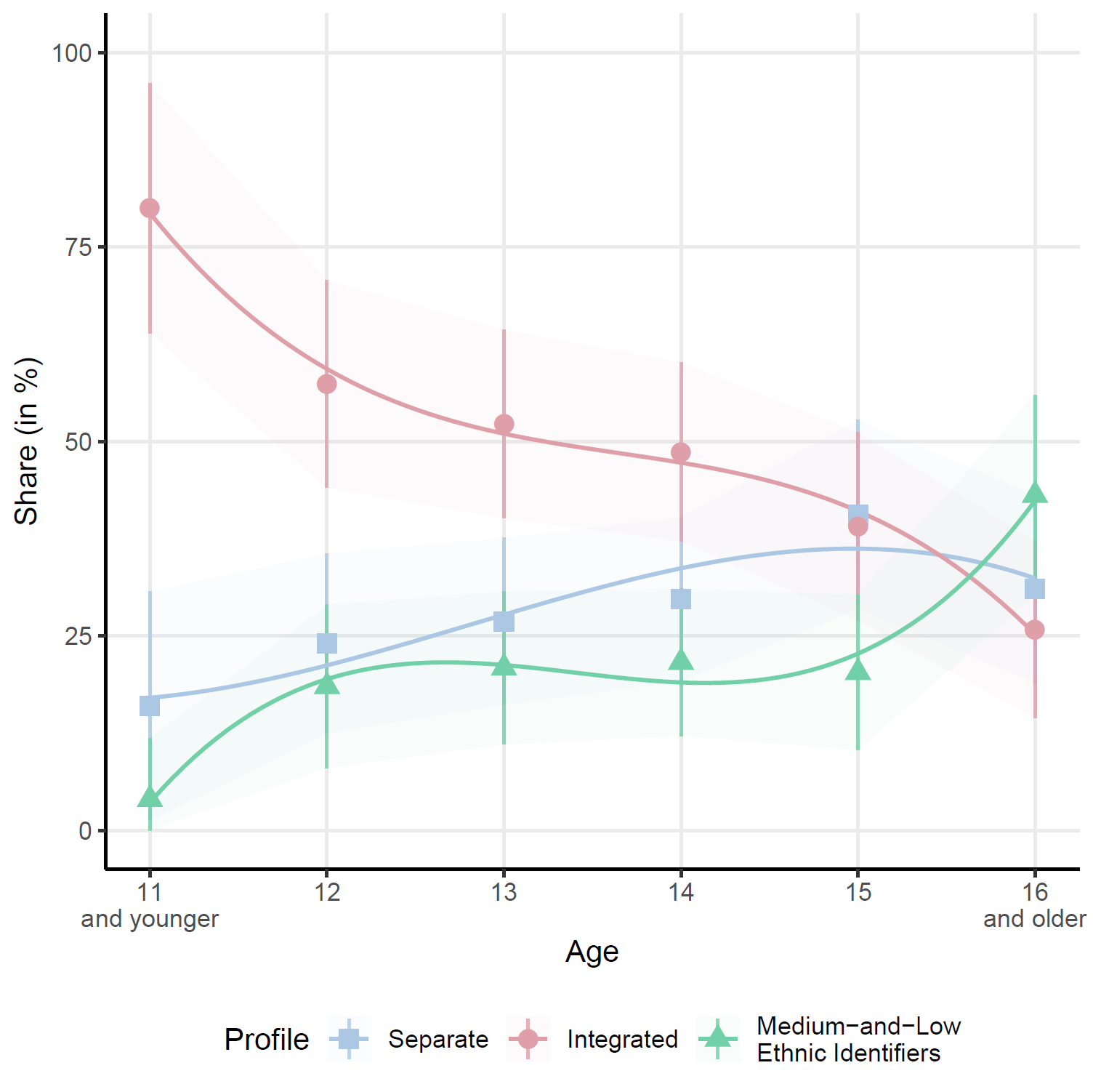 |

# Part B

## Table B1. Latent Class Analysis Model Selection for All Five Waves for the Full Sample of Students with Migration Background

| Classes | Goodness of Fit | | | Size (Share in %) | | | |
| --- | --- | --- | --- | --- | --- | --- | --- |
|  | BIC | BLRT^a^ | Entropy | Class 1 | Class 2 | Class 3 | Class 4 |
| *Wave 1* (N = 685) | | | | | | | |
| 2 | 26149.6 | 1850.4 | 0.92 | 17.5 | 82.5 |  |  |
| 3 | 24819.8 | 1413.3 | 0.90 | 17.7 | 27.3 | 55.0 |  |
| 4 | 24244.4 | 666.5 | 0.91 | 29.2 | 20.7 | 3.2 | 46.9 |
| *Wave 2* (N = 783) | | | | | | | |
| 2 | 29989.0 | 2427.3 | 0.92 | 20.7 | 79.3 |  |  |
| 3 | 28376.7 | 1695.3 | 0.90 | 17.2 | 25.0 | 57.7 |  |
| 4 | 27525.2 | 942.0 | 0.92 | 23.9 | 23.3 | 4.0 | 48.8 |
| *Wave 3* (N = 820) | | | | | | | |
| 2 | 31665.2 | 3008.3 | 0.93 | 21.1 | 78.9 |  |  |
| 3 | 29969.3 | 1796.6 | 0.90 | 19.4 | 29.9 | 50.7 |  |
| 4 | 28935.4 | 1134.6 | 0.92 | 27.3 | 26.3 | 4.0 | 42.4 |
| *Wave 4* (N = 814) | | | | | | | |
| 2 | 31557.7 | 3049.0 | 0.92 | 25.7 | 74.3 |  |  |
| 3 | 30003.7 | 1654.5 | 0.90 | 23.5 | 47.5 | 29.0 |  |
| 4 | 28884.4 | 1219.8 | 0.92 | 24.5 | 31.0 | 6.5 | 38.0 |
| *Wave 5* (N = 766) | | | | | | | |
| 2 | 28913.9 | 3115.4 | 0.93 | 24.9 | 75.1 |  |  |
| 3 | 27394.6 | 1602.9 | 0.91 | 24.0 | 27.7 | 48.3 |  |
| 4 | 26167.9 | 1313.1 | 0.92 | 26.4 | 30.4 | 5.9 | 37.3 |
| *Note*: ^a^ Differences between the BLRT of a model with one more class versus one fewer class were significant at p < 0.001 for all waves and all 15 model comparisons. | | | | | | | |

## Table B2. LCA Descriptive Means for Students with Migration Background for First Wave (N = 685)

|  | Separated | Integrated | Low- and Medium-  Ethnic Identifiers |
| --- | --- | --- | --- |
| Female (in %; Range 0-100) | 51.9 | 51.2 | 50.4 |
| Age (Range 10.1-16.8) | 12.79 ^a^ | 12.53 ^b^ | 12.61^a,b^ |
|  | (1.13) | (1.08) | (1.03) |
| Academic year (Range 5-7) | 6.12^a^ | 5.93^b^ | 6.12^a^ |
|  | (.82) | (.85) | (.82) |
| Self-categorization as one from my | 3.92^a^ | 3.21^b^ | 2.56^c^ |
| family’s country of origin (Range 1-5) | (1.01) | (1.05) | (1.03) |
| Dual identification (Range 1-5) | 3.25 ^a^ | 4.07 ^b^ | 3.77 ^c^ |
|  | (1.60) | (1.30) | (1.20) |
| Impermeability (Range 1-5) | 2.99 ^a^ | 3.02 ^a^ | 2.63 ^b^ |
|  | (1.25) | (1.16) | (1.13) |
| Liking Germans (Range 1-5) | 3.86 ^a^ | 4.59 ^b^ | 4.55 ^b^ |
|  | (1.09) | (.65) | (.73) |
| GPA (Range 1-6) | 3.12 ^a^ | 2.92 ^b^ | 2.72 ^c^ |
|  | (.72) | (.78) | (.82) |
| Turkish Ethnic Origin (in %; Range 0-100) | 42.2 ^a^ | 38.5 ^a^ | 21.5 ^b^ |
| Non-believers (in %; Range 0-100) | 13.6 ^a^ | 14.4 ^a^ | 28.9 ^b^ |
| Christian (in %; Range 0-100) | 32.6 ^a^ | 40.4 ^b^ | 50.4 ^b^ |
| Muslim (in %; Range 0-100) | 53.8 ^a^ | 45.0 ^b^ | 20.7 ^c^ |
| German friends (in %; Range 0-100) | 23.5 ^a^ | 28.4 ^b^ | 40.6 ^c^ |
|  | (24.5) | (26.3) | (29.9) |
| *Note*: Means with different superscripts significantly different at *p* < .05 | | | |

## Table B3. Transition Probabilities for Students with Migration Background (N = 1,023)

|  | Wave 3 | | | Wave 5 | | |
| --- | --- | --- | --- | --- | --- | --- |
|  | Separated  (31.57%) | Integrated  (47.70%) | Medium- and Low-Ethnic  (20.73%) | Separated  (30.11%) | Integrated  (46.04%) | Medium- and Low-Ethnic  (23.85%) |
| Wave 1  Separated (23.56%) | 0.64 | 0.26 | 0.10 | 0.60 | 0.28 | 0.12 |
| Integrated (62.95%) | 0.22 | 0.63 | 0.15 | 0.15 | 0.69 | 0.16 |
| Medium‑ and Low-Ethnic (13.49%) | 0.15 | 0.21 | 0.64 | 0.15 | 0.13 | 0.72 |

## Table B4. Transition Probabilities Moderated by Discrimination for Students with Migration Background (N = 1,016)

|  | *OR* | *SE* |  | *p-value* |
| --- | --- | --- | --- | --- |
| *Effect of perceived discrimination on profile attribution* | | | | |
| *Wave 1 attribution of profile* |  |  |  |  |
| Separate | 1.132 | 0.132 |  | 0.316 |
| Integrated (Reference) | 1 |  |  |  |
| Medium- and Low-Ethnic Identifiers | 0.647 | 0.117 | ** | 0.003 |
| *Wave 3 attribution of profile* |  |  |  |  |
| Separate | 0.913 | 0.149 |  | 0.561 |
| Integrated (Reference) | 1 |  |  |  |
| Medium- and Low-Ethnic Identifiers | 1.201 | 0.217 |  | 0.356 |
| *Effect of perceived discrimination on profile transitions between waves* | | | | |
| *Wave 1 to Wave 3* |  |  |  |  |
| Separate (in wave 1) |  |  |  |  |
| to Separate (in wave 3) | 1.066 | 0.293 |  | 0.821 |
| to Integrated (in wave 3; Reference) | 1 |  |  |  |
| to Medium- and Low-Ethnic Identifiers (in wave 3) | 0.809 | 0.336 |  | 0.569 |
| Integrated (in wave 1) |  |  |  |  |
| to Separate (in wave 3) | 1.092 | 0.217 |  | 0.672 |
| to Integrated (in wave 3; Reference) | 1 |  |  |  |
| to Medium- and Low-Ethnic Identifiers (in wave 3) | 0.718 | 0.173 |  | 0.102 |
| Medium- and Low-Ethnic (in wave 1) |  |  |  |  |
| to Separate (in wave 3) | 1.284 | 0.850 |  | 0.738 |
| to Integrated (in wave 3; Reference) | 1 |  |  |  |
| to Medium- and Low-Ethnic Identifiers (in wave 3) | 0.507 | 0.271 |  | 0.069 |
| *Wave 3 to Wave 5* |  |  |  |  |
| Separate (in wave 3) |  |  |  |  |
| to Separate (in wave 5) | 1.119 | 0.261 |  | 0.650 |
| to Integrated (in wave 5; Reference) | 1 |  |  |  |
| to Medium- and Low-Ethnic Identifiers (in wave 5) | 0.432 | 0.363 |  | 0.118 |
| Integrated (in wave 3) |  |  |  |  |
| to Separate (in wave 5) | 1.343 | 0.310 |  | 0.269 |
| to Integrated (in wave 5; Reference) | 1 |  |  |  |
| to Medium- and Low-Ethnic Identifiers (in wave 5) | 0.868 | 0.224 |  | 0.555 |
| Medium- and Low-Ethnic (in wave 3) |  |  |  |  |
| to Separate (in wave 5) | 0.824 | 0.357 |  | 0.622 |
| to Integrated (in wave 5; Reference) | 1 |  |  |  |
| to Medium- and Low-Ethnic Identifiers (in wave 5) | 0.717 | 0.284 |  | 0.319 |

## Figure B1. Ethnic and National Identification across Profiles at all Waves for the Full Sample of Students with Migration Background


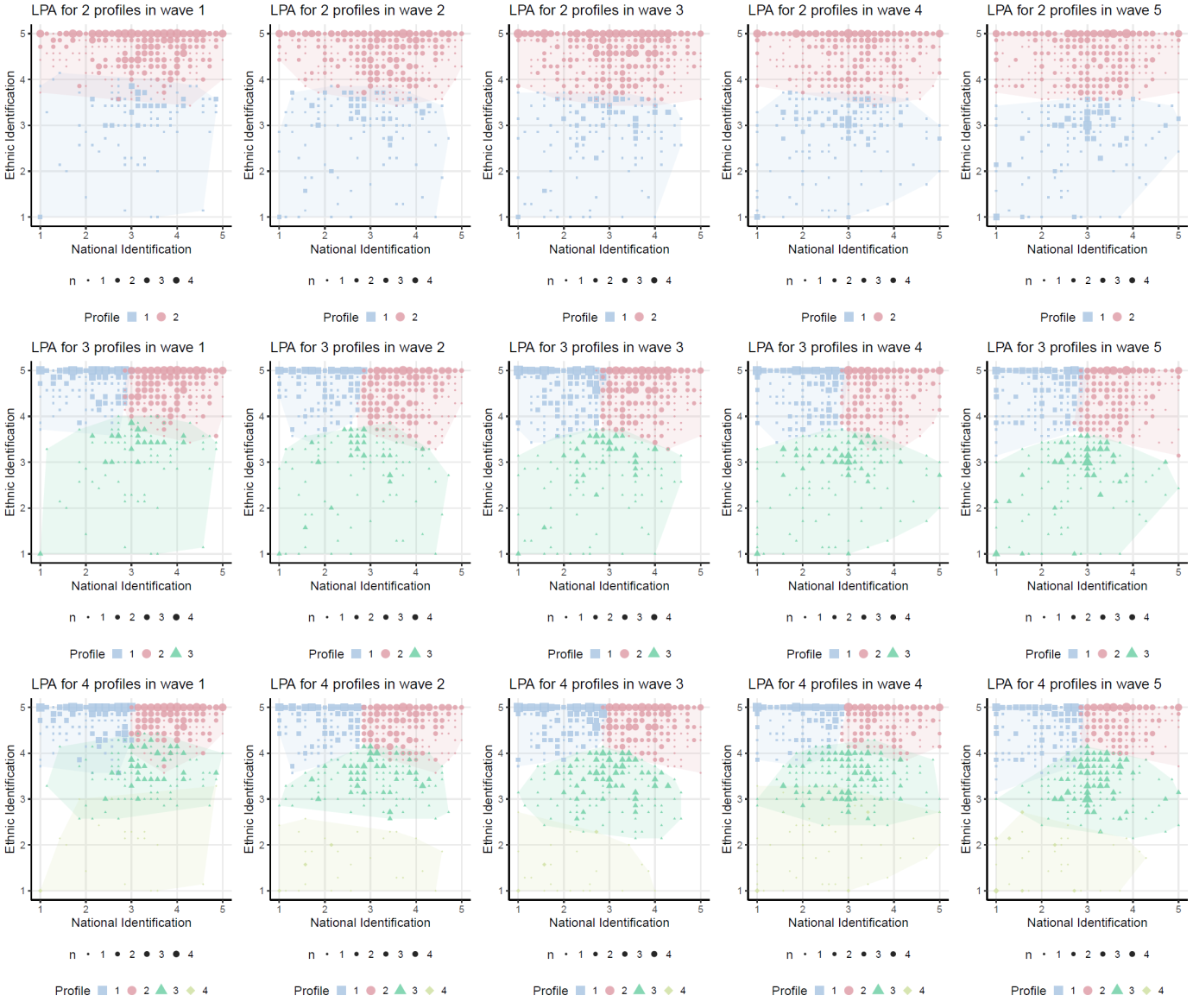


## Figure B2. Profile Shares across the Observation Period


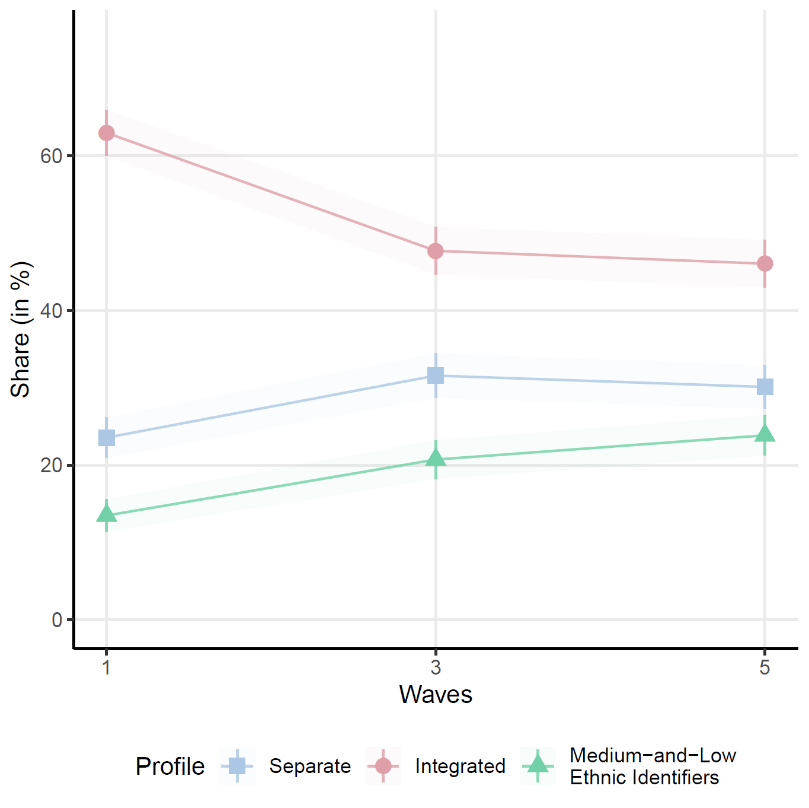


## Figure B3. Profile Shares Across Different Ages


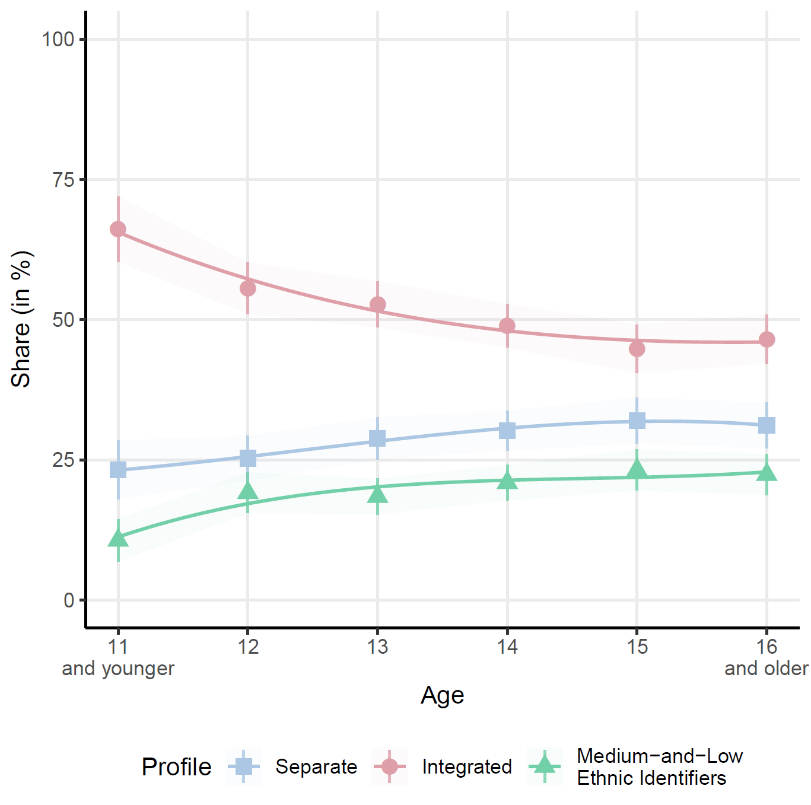

Supplement: Supplementary file 1 — Supplementary Materials [file 10964_2020_1250_MOESM1_ESM.docx]
